# In Silico Investigation of Parkin-Activating Mutations Using Simulations and Network Modeling

**DOI:** 10.3390/biom14030365

**Published:** 2024-03-19

**Authors:** Naeyma N. Islam, Caleb A. Weber, Matt Coban, Liam T. Cocker, Fabienne C. Fiesel, Wolfdieter Springer, Thomas R. Caulfield

**Affiliations:** 1 Department of Neuroscience, Mayo Clinic, 4500 San Pablo Road, Jacksonville, FL 32224, USA; islam.naeyma@mayo.edu (N.N.I.); weber.caleb@mayo.edu (C.A.W.); coban.mathew@mayo.edu (M.C.); fiesel.fabienne@mayo.edu (F.C.F.); 2 Neuroscience PhD Program, Mayo Clinic Graduate School of Biomedical Sciences, 4500 San Pablo Road, Jacksonville, FL 32224, USA; 3 Department of Neurosurgery, Mayo Clinic, 4500 San Pablo Road, Jacksonville, FL 32224, USA; 4 Department of Cancer Biology, Mayo Clinic, 4500 San Pablo Road, Jacksonville, FL 32224, USA; 5 Department of Biochemistry & Molecular Biology, Mayo Clinic, 4500 San Pablo Road, Jacksonville, FL 32224, USA; 6 Department of Computational Biology, Mayo Clinic, 4500 San Pablo Road, Jacksonville, FL 32224, USA

**Keywords:** Parkinson’s disease, parkin, network modeling, molecular dynamics simulation

## Abstract

Complete loss-of-function mutations in the PRKN gene are a major cause of early-onset Parkinson’s disease (PD). PRKN encodes the Parkin protein, an E3 ubiquitin ligase that works in conjunction with the ubiquitin kinase PINK1 in a distinct quality control pathway to tag damaged mitochondria for autophagic clearance, i.e., mitophagy. According to previous structural investigations, Parkin protein is typically kept in an inactive conformation via several intramolecular, auto-inhibitory interactions. Here, we performed molecular dynamics simulations (MDS) to provide insights into conformational changes occurring during the de-repression of Parkin and the gain of catalytic activity. We analyzed four different Parkin-activating mutations that are predicted to disrupt certain aspects of its auto-inhibition. All four variants showed greater conformational motions compared to wild-type protein, as well as differences in distances between domain interfaces and solvent-accessible surface area, which are thought to play critical roles as Parkin gains catalytic activity. Our findings reveal that the studied variants exert a notable influence on Parkin activation as they alter the opening of its closed inactive structure, a finding that is supported by recent structure- and cell-based studies. These findings not only helped further characterize the hyperactive variants but overall improved our understanding of Parkin’s catalytic activity and nominated targets within Parkin’s structure for potential therapeutic designs.

## 1. Introduction

Parkinson’s disease (PD) is the most prevalent neurodegenerative movement disorder, affecting millions all over the world [1]. The majority of instances of early-onset PD are caused by mutations in the genes PRKN and PINK1 [2,3]. The PRKN gene encodes the protein Parkin, an E3 ubiquitin (Ub) ligase that mediates a mitochondrial quality control pathway. This system is also dependent on the activation of the Ub kinase PINK1 [4,5,6,7]. Upon mitochondrial membrane depolarization, PINK1 is stabilized on damaged organelles and phosphorylates existing Ub molecules at Ser65 [8,9,10]. p-S65-Ub plays a crucial role in the activation and recruitment of auto-inhibited Parkin from the cytosol and is followed by the phosphorylation of Parkin itself at the conserved Ser65 residue in its N-terminal Ub-like (UBL) domain [11,12,13]. Once fully activated, Parkin labels multiple mitochondrial proteins with additional Ub moieties, which are then further phosphorylated by PINK1 in a positive feedback loop. The decoration of mitochondria with p-S65-Ub by the concerted actions of PINK1 and Parkin is thought to facilitate their selective degradation via the autophagy–lysosome system (termed mitophagy) [14].

Structural investigations have revealed fundamental information regarding Parkin activation at the molecular level. Parkin is a member of the RING-in-Between-RING (RBR) family of E3 Ub ligases [15]. It transfers Ub to a substrate from an E2 Ub-conjugating enzyme by forming a thioester intermediate on a reactive cysteine (Parkin C431) in the RING2 catalytic domain. Notably, Parkin is auto-inhibited in the basal, unstimulated state, according to structural and biochemical analysis [16,17,18,19,20]. However, it has been postulated that the activation of Parkin’s enzymatic functions and its mitochondrial translocation are linked [5,7,21,22,23].

In Parkin’s auto-inhibited state, the E2 binding site on RING1 is blocked by the UBL domain and the Repressor Element of Parkin (REP), and the catalytic center C431 is trapped at the interface of RING0 and is, thus, too far from the E2~Ub conjugate for effective transfer. For the activation of Ub ligase activity, both C431 and the E2 binding site must become accessible. Following the activation of PINK1, upon binding to p-S65-Ub, the UBL domain is freed from RING1, itself becoming available for phosphorylation at S65 by PINK1 [7,24,25,26,27]. Phospho-UBL and the ACT (activating) element subsequently attach to RING0, resulting in disruption of the RING2-RING0 interface and releasing the catalytic center C431, coinciding with the release of the REP from RING1, allowing for E2~Ub binding and ubiquitination of outer mitochondrial membrane proteins [28,29,30,31].

According to the findings of the investigations conducted in vitro, interdomain interactions are what keep Parkin in an auto-inhibited state. Indeed, mutations of residues located at inhibitory interfaces, such as F146 in RING0 or W403 in the REP, result in a dramatic increase in the E3 Ub ligase activity of Parkin and its mitochondrial recruitment [32]. Furthermore, these synthetic mutations, such as F146A and W403A, can rescue S65A or ΔUBL Parkin mutations in ubiquitylation and mitophagy assays [25,33,34,35]. The resulting de-repression of Parkin is even able to rescue seven PD-associated Parkin missense mutations [36]. These mutations affect Parkin activity through a variety of different mechanisms. However, the N273K mutation in RING1, which is responsible for the repulsion of the UBL domain, speeds up the recruitment of Parkin to mitochondria but does not rescue the S65A mutation with regards to Parkin recruitment or substrate ubiquitylation [35]. Counterintuitively, strongly activating mutations may cause rapid Parkin turnover via auto-ubiquitylation due to constitutive activity in the cytosol. Such self-regulation is known for many activated E3 Ub ligases [37] and is further, although indirectly, supported by a noticeable increase in Parkin protein in the absence of the activating kinase PINK1 [38]. As such, the overactivation of Parkin may ultimately lower the protein’s steady-state levels in cells and consequently lower its actual cellular activity toward mitochondrial substrates. Therefore, such mutations have the potential to adjust Parkin’s activity and stability to varying degrees. As a result, a systematic and complete evaluation of activating mutations is essential. Recent findings regarding the structural, biochemical (thermal stability), and functional assays on mutations V393D, A401D, and W403A rescuing a Parkin S65A mutant demonstrate that the destabilization of the RING0:RING2 or REP:RING1 interfaces would promote alterations in activity of the enzyme that may be beneficial [39].

## 2. Materials and Methods

### 2.1. Model Construction

Since no complete experimental structure for human Parkin exists, gaps in the structures were completed using the sequence UniProt accession #O60260. The final model consists of residues 1–465 of human Parkin, with eight zincs bound. The final model was generated using homology modeling with YASARA [40], in a similar manner as described in our previous study [21]. After the construction of the original model, variants were generated by the mutagenesis wizard in PyMOL. We compared our homology model to the model predicted by AlphaFold; despite being a newer algorithm, we believe it resulted in an inferior model, with some commentary given in the discussion section.

### 2.2. Molecular Dynamics Simulations

We conducted identical all-atom unbiased molecular dynamics simulations (MDS) on wild-type (WT) Parkin and several published variants known to promote Parkin activation (Y143E, V393D, A401D, and W403A) [39]. MDS were carried out using techniques (e.g., YASARA, Amber) [40], which have been previously described [41,42]. We compared our results against other simulators for any defects, which included brief NAMD2 and Desmond relaxation runs. These simulations were performed to investigate the impact of the variants on the conformational dynamics of Parkin and determine the mechanistic etiology of hyperactivity. In brief, each Parkin variant was subjected to energy minimization with relaxed restraints using the steepest descent *Polak-Ribiere* conjugate gradient method [43]. The simulation box was constructed 18 Å from the nearest protein atom and then filled with TIP3P waters at 0.997 g/L, Na^+^/Cl^-^ at 150 mM, 310 K, 1 bar, pH 7.4, while using an Amber force field within YASARA with long-range Coulombic forces that were calculated using Particle-Mesh-Ewald periodic boundary conditions at a cutoff of 7.86 Å. The final system sizes were approximately 207,000 atoms, of which 200,000 atoms were explicit waters. The NPT thermodynamic ensemble was utilized for analytical simulations; the temperature was maintained by a Berendsen weak coupling thermostat [44]. The pressure was maintained by the Parrinello–Rahman Barostat algorithm [45]. The simulation timestep was 2.5 fs. Following relaxation and equilibration, simulations were carried out for the purpose of analysis. Combined simulations completed a total of over 2 microseconds across all variants.

### 2.3. Analyses of Simulation Data

MDTraj was used to perform an assessment of the root mean square deviation (RMSD) and root mean square fluctuation (RMSF) calculations [46]. The nearest-neighbor function of MDTraj was utilized to calculate any residue that is located within 5 Å from the RING1 binding site. MDTraj employs the Shrake–Rupley algorithm to calculate the solvent-accessible surface area (SASA) of individual residues within the Parkin protein. Visual Molecular Dynamics (VMD) was employed for the purpose of computing the center-of-mass distance between distinct domains [47]. PyMOL and BioLuminate were used for visualization and structure comparisons [48,49].

Overall, these molecular dynamics modeling techniques, methods, analyses and the development of these tools have been described in the literature [50,51,52,53,54].

## 3. Results

In this study, we have incorporated mutations V393D, A401D, and W403A on the REP domain, as well as the Y143E mutation on RING0, and evaluated their effects on Parkin’s activity by analyzing conformational changes between different domains in comparison to WT Parkin using a recognized extended molecular dynamics simulation.

### 3.1. Stability and Flexibility (RMSD and RMSF)

**Figure 1:**
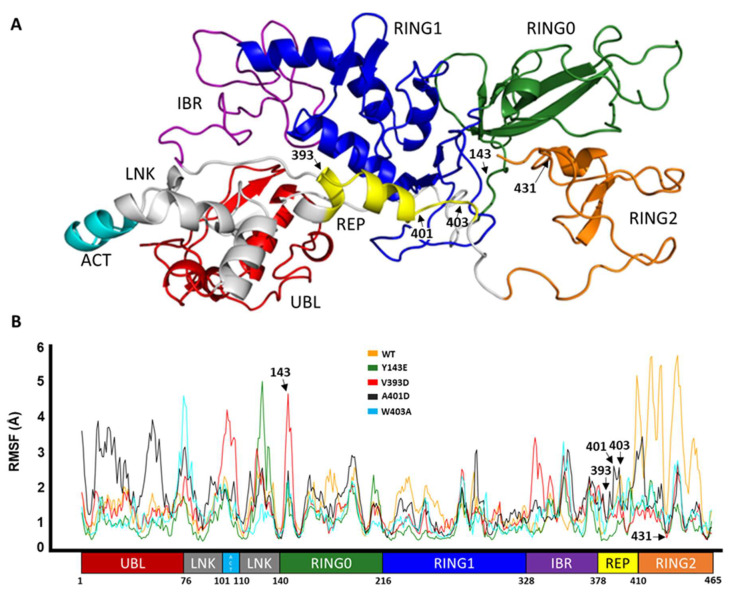
Domain representation of Parkin. (**A**) Domain color-coded onto the 3D structure of Parkin. (**B**) Cα RMSF of Parkin WT and activating variants, with the domain-based residue numbering for context.

Parkin is composed of several distinct domains including UBL, LNK, ACT, RING0, RING1, IBR, REP, and RING2 domains, and these domains must undergo conformational changes that open up its structure and allow access to its catalytic site, C431 [29]. The domain map of Parkin is given in Figure 1A. Here, we will discuss how conventional molecular dynamics studies (MDS) shed light on the structural dynamics of the different domains of Parkin as a result of different mutations by capturing its early conformational changes, therefore improving our understanding of its activation.

#### 3.1.1. Root Mean Square Fluctuation Analyses Demonstrate Altered Domain Flexibility of Parkin-Activating Mutations

Here we show the corresponding effect of the activating mutation on the fluctuation in the variants versus the wild type along the entire length of Parkin protein as a consequence of the simulation.

RMSF is used to represent how much a particular residue fluctuates during a simulation and can indicate which residues/domains are contributing the most to a molecular motion. Figure 1B displays the RMSF values for the C-alpha atoms of different domains of Parkin. The UBL domain of A401D Parkin shows the highest fluctuation as shown by its RMSF value of 4 Å, compared to the other mutations and WT Parkin. In contrast to both, mutations Y143E, V393D, and W403A show more fluctuation in and adjacent to the linker region but less motion within the RING2 domain, especially in comparison to WT Parkin. Overall, the domains seem to have become more flexible with each of the mutations, except the RING2 domain has undergone a more substantial transformation in WT than the variants.

#### 3.1.2. Root Mean Square Deviation Analyses of ‘Open’ Parkin Structures Suggest an Increased Deviation from the ‘Closed’ (Native) Structure for Each of the Hyperactive Variants

**Figure 2:**
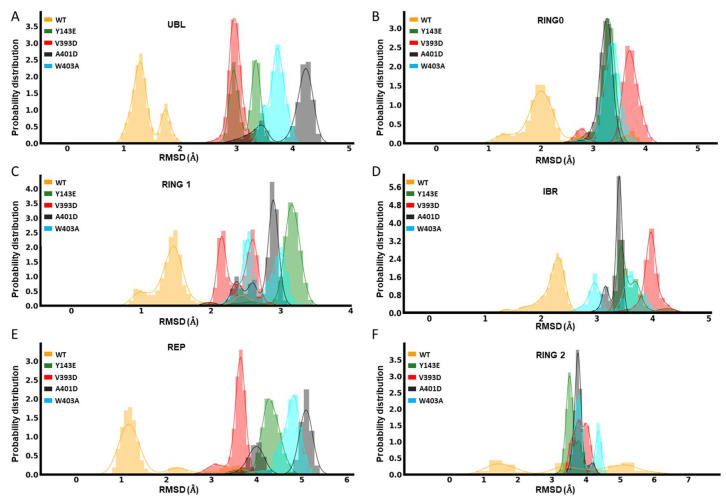
Domain-based dynamical variation comparisons. Domain-level RMSD of Parkin variants Y143E, V393D, A401D, W403A, and WT: UBL (**A**), RING0 (**B**), RING1 (**C**), IBR (**D**), REP (**E**), and RING2 (**F**).

The opening of Parkin is dependent on the structural deviation of its native closed structure. This structural deviation can be thought of as a key regulator of Parkin’s activity. The UBL domain works like a spring and clamp to keep Parkin in its closed shape. Comparisons of the root mean square deviation (RMSD) in atomic positions of the UBL domain revealed there is less deviation observed with the WT UBL structure than with the different mutants (Figure 2A). We can, therefore, infer that the hyperactive mutants have a less robust UBL clamp and are thus suspected to be more freely activatable. We also found that the RMSD of other domains, such as RING0 (Figure 2B), RING1 (Figure 2C), IBR (Figure 2D), and REP (Figure 2E), is significantly larger for mutants compared to WT Parkin. The mutation Y143E causes the most substantial deviation for RING1, with an RMSD of 3.5 Å. Whereas, as shown in Figure 2B, the V393D mutation causes the most deviation in the RING0 domain, while the mutation A401D causes the most significant divergence for the REP and UBL domains. The RMSD values for the REP, UBL, and RING1 domains are also significantly increased by 5.3 Å, 4.19 Å, and 3.31 Å, respectively, with the W403A mutation. The catalytic domain RING2 is also expected to undergo distinct conformational changes during activation [28]. The RMSD values for the RING2 were also calculated for mutant and WT Parkin (Figure 2F). Here, we found there is considerable structural deviation from the native (closed) structure for both WT and variants, but this appears much more pronounced for the activating mutants, converging at 3.5–4.5 Å in comparison to WT, which shows a much more varied set of RMSD values (0.5–6.5 Å).

While not as telling as individual domain RMSD values, the global RMSD changes between different mutations and WT for the entire Parkin protein are also shown in Figure S1, highlighting V393D and A401D as notably more dissimilar from the native structure compared to the two other variants or WT Parkin.

During the 400 nanosecond (ns) simulations, the higher RMSD and RMSF values for each of the mutations demonstrated more pronounced structural changes compared to the WT Parkin structure. Most notably, the highest fluctuation for the RING2 domain is seen with WT Parkin of 5–6 Å (RMSD), in stark contrast to the hyperactive variants which demonstrate much less molecular motion around this domain. This suggests the RING2 domain may play a more important role in the early stages of activation than was previously indicated by static structures, as this region differs when compared to hyperactive variants, which have been previously shown to increase activity in vitro and in vivo.

#### 3.1.3. Dynamical Network Analyses Identify Variant-Specific Changes in Residue Algorithm-Assigned Communities

**Figure 3:**
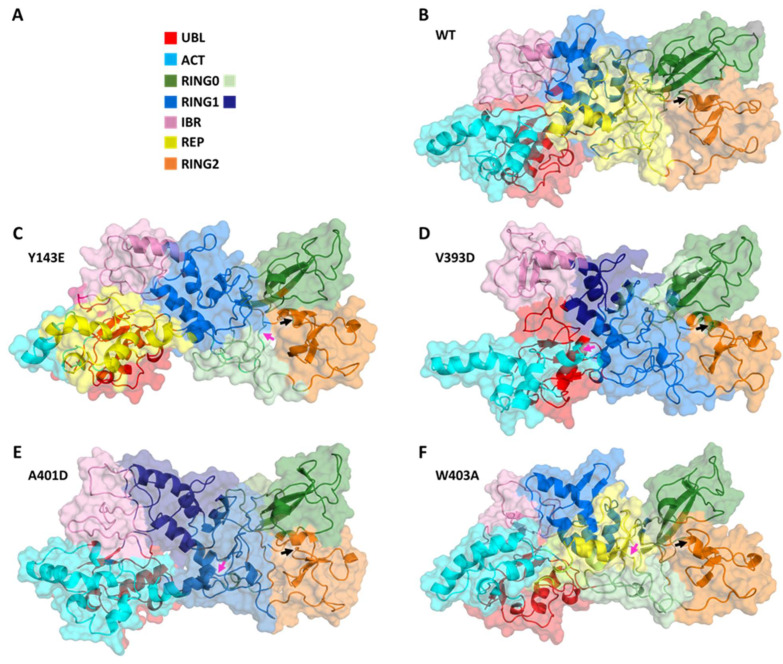
Dynamical network comparisons of Parkin variants. (**A**) Legend for community colorations, organized by Parkin structural domain. Some domains in some variants belong to more than one community and are given a second color. (**B**–**F**) Community maps for Parkin variants. (**B**) WT, (**C**) Y143E, (**D**) V393D, (**E**) A401D, and (**F**) W403A. Variant residue indicated with a magenta arrow and C431 indicated with a black arrow.

Given the differences in global dynamical profiles and conformations, we hypothesized that the variants altered local non-bonded interaction connectivity and, thus, kinetic information transmission pathways. We performed dynamical network analysis using the Girvan–Newman algorithm [55] and analyzed network-based statistics [56]. The algorithm defines each residue as a node and draws edges corresponding to non-covalent interactions (e.g., hydrogen bonds) that occur throughout the course of the simulation. Residues are assigned to communities where kinetic energy (i.e., motion) is shared via strong non-covalent connectivity. Kinetic energy and motion within each community can be more easily propagated than motion across multiple communities of the protein. Figure 3 displays how each variant impacts the protein’s dynamical community organization. Each colored region represents a different community within the structural network. WT Parkin’s REP domain has a community that roots itself deep into the core structure of the protein, overlapping with domains RING0 and RING1 (Figure 3). Each variant consistently disrupts this anchoring effect, often splitting the REP domain into multiple communities. This is likely to negatively impact the REP’s ability to relay kinetic information to the protein as a whole. Furthermore, we notice that in most instances (Y143E, V393D, and A401D) we see the community containing the activation (ACT) element (residues 101–109) has a much stronger influence over the REP region. This too could impact the activation process by allowing the ACT domain to more easily relay the kinetic information required to start the activation process. These data suggested interdomain interactions would reveal mechanistic insight into the initiation of activation.

### 3.2. Interdomain Center-of-Mass (COM-COM) Distances Measure an Increased Gap between the UBL and RING1 in Hyperactive Variants

We measured the center-of-mass distance between the UBL and RING1 domains as a metric to assess how far apart the UBL domain is from the RING1 domain, which is required to loosen the entire structure.

**Figure 4:**
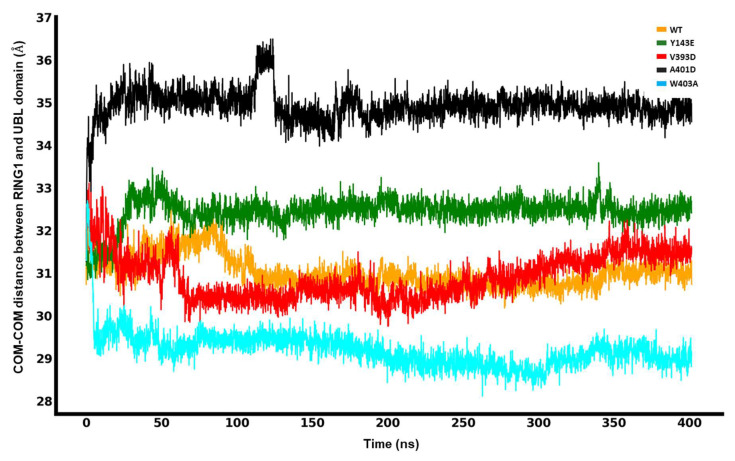
Center-of-mass (COM-COM) distances between the RING1 and UBL domains for different mutations and WT Parkin.

The molecular dynamics simulation data, presented in Figure 4, elucidates the spatial relationship between the UBL and RING1 domains for the different mutations and the wild type. For the W403A mutation, the minimum interdomain COM-COM distance was approximately 29 Å, indicative of a close interaction. The V393D mutation displayed a trend of increasing distance, whereas the A401D mutation showed a significant shift in the COM distance around the 100 ns mark, stabilizing at about 35.5 Å for the remainder of the simulation. The Y143E mutation maintained a relatively stable distance of 33 Å. In comparison, the W403A mutation exhibited the shortest COM distance, and the A401D mutation had the longest, with the order of increasing COM distance being W403A < WT ≈ V393D < Y143E < A401D. It is important to note that the simulation is not intended to capture the complete dissociation of the UBL domain from RING1 but the factors that trigger or frustrate it from starting.

### 3.3. Release of the REP Region from RING1

A key step in Parkin activation involves engagement with an Ub-charged E2 co-enzyme (E2~Ub). In the absence of PINK1 activation, Parkin is kept in a closed, auto-inhibited conformation. Therefore, to provide room for the structural rearrangements required for the binding of E2~Ub, the REP region first needs to be released from RING1, which involves the disruption of intermolecular interactions between the two. During the simulation (400 ns), we computed the likelihood that any given REP residue would be found less than 5 Å away from the E2 binding site on RING1 to evaluate the stability of the intermolecular interaction between REP and RING1 for each different mutation and WT Parkin.

**Figure 5:**
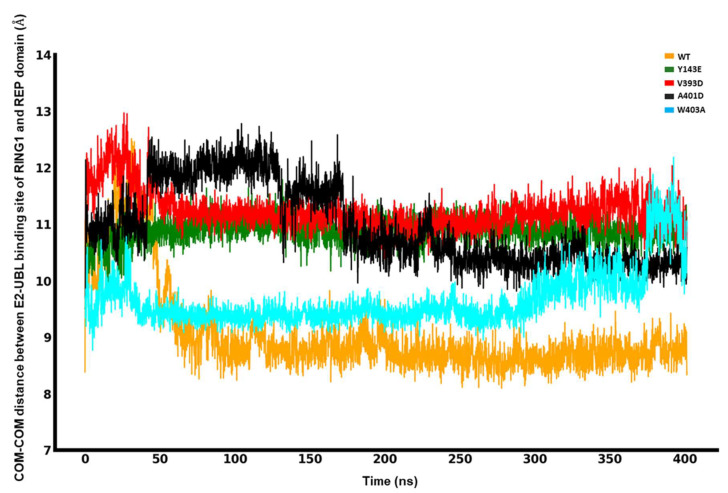
Center-of-mass (COM-COM) distances between the RING1 and REP domains for different mutations and WT.

The distance between REP and RING1 is particularly important because it can indicate the release of the REP region from the E2 binding site in RING1. For this purpose, we calculated the center-of-mass distance between the E2 binding site on RING1 and REP. Our MDS analyses reveal, as shown in Figure 5, that for the WT, the distance between REP and the E2 binding site goes from a minimum of 8.09 Å to a maximum of 12.51 Å. However, this rapid increase is not constant, as it significantly drops back to 9.06 Å and remains around this level for the rest of the simulation (75% of the time in the simulation). Over the first 180 ns of the simulation, the distance between REP and the E2 binding site for the A401D mutation increased from 9.7 Å to a maximum of 12.78 Å and then stabilized at a value of 11.65 Å, a constancy that was maintained throughout the remaining 75% of the total simulation duration. Notably, this distance is consistent 75% of the time at 11.04 Å and 11.36 Å from a minimum of 9.51 Å and 9.70 Å for the Y143E and V393D mutations, respectively. Hence, it is plainly visible that REP dissociates from the E2 binding site for the least amount of time for WT compared to the mutated forms of Parkin, describing an altered interaction between the REP and RING1 in the hyperactive variants, which keeps the site accessible for E2~Ub for longer. Even though our established MDS does not capture the full dissociation of the REP, there is sufficient indication of trends in the corresponding conformational rearrangement that exposes the E2 binding site of RING1 to allow access for an incoming E2~Ub complex to the binding site. These findings will help bridge the gap between Parkin’s static structure and conformational dynamics to better understand its enzymatic functions that result from different mutations.

**Figure 6:**
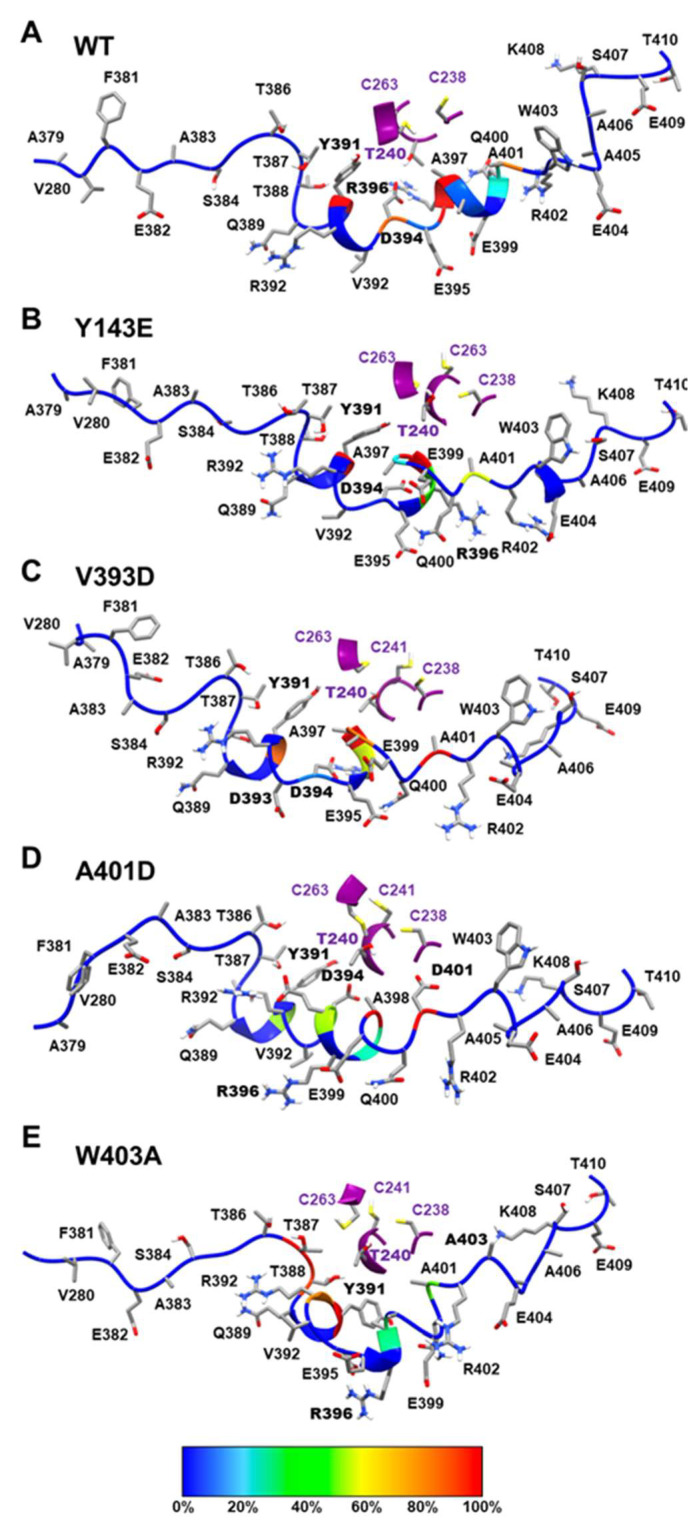
Binding interaction between E2 binding site formed with RING1 and REP. Our binding interaction analysis is based on the presence of REP residues that are under a 5 Å distance from the E2 binding site during the 400 ns simulation. The REP backbone is displayed in different colors based on their time occupancy from simulations. This is determined by their relative proximity to the RING1 region, where red represents the 90–100% occupancy and blue represents the 0% occupancy of REP residue. (**A**) WT region around REP-RING1, similarly shown (**B**) Y143E mutant, (**C**) V393D mutant, (**D**) A401D mutant, and (**E**) W403A mutant.

We can utilize the proximity of the E2 binding site on RING1 with the REP element as a readout for Parkin repression. This is made simpler by looking at the interaction between various residues belonging to the REP with a specific residue of the E2 binding site on RING1 (e.g., T240). The longer these interactions last throughout the simulation, the more frozen the inactive state Parkin is, and we expect this to be the opposite for the hyperactive variants. Using a color key, we colored the REP residue in accordance with their finding at 5 Å from the E2 binding site. Figure 6 and  Figure S3 show that for the WT, T240 (E2 binding site) forms a stable interaction with R396 (REP) that lasts throughout the simulation (100% of the time) and indicates a strong binding between them. However, this binding is disrupted, displaying a reduced interaction for V393D (70%) and Y143E (40%) mutations, and it is completely absent for A401D (0%) and W403A (0%) mutations (Figure 6 and  Figure S3). The interaction between T240 (E2 binding site) and Y391 (REP) is also highly unstable for A401D (~50%), whereas it is detected consistently for WT (100%) along with W403A (100%) and Y143E (100%). All percentages shown refer to the amount of simulation time. Another notable interaction seen with WT Parkin is the H-bond formed between T240 (E2 binding site on RING1) and D394 (REP) in 95% of the time, which is not found in any of the four proposed mutations and helps further elucidate the structural mechanisms of hyperactivity in each of the variants.

To activate Parkin, as well as releasing REP from RING1, RING0 must be released from RING2 to make the catalytic center C431 accessible. The interaction between RING0 and RING2, and RING0 and RING1 is illustrated in terms of energy in Figures S4 and S5, respectively, for the various mutations (Y143E, V393D, A401D, and W403A), and WT. These activating mutations appear to differentially alter the RING0-RING2 interaction energy. All variants located in the REP region result in an increase in this interaction, the greatest increase seen with V393D. In contrast, Y143E, a mutation that resides within the RING0 domain showed a decrease in this interaction. Notably, all mutations have been previously shown to activate Parkin in vitro (all comparable to W403A) [39].

### 3.4. Analysis of RING2 Catalytic Residue C431

#### 3.4.1. Solvent-Accessible Surface Area of C431

Hydration of the cavity surrounding C431 indicates the availability to receive Ub [21]. For this purpose, we calculated the solvent-accessible surface area (SASA) of C431 for each of the different mutations and the WT. It is worth mentioning that the RING0 domain covers this catalytic cysteine on the RING2 domain—to make the C431 accessible to solvent, RING0 must be removed. However, our findings did not show any significant changes in the center-of-mass distances between RING0 and RING2, perhaps because this traditional MD simulation could not capture the whole conformational opening. Therefore, this could potentially affect the SASA.

**Figure 7:**
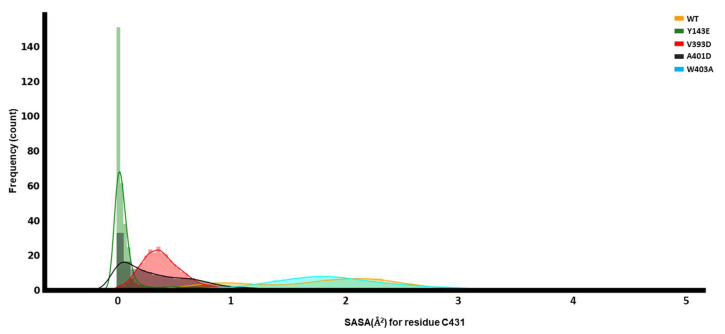
Dynamical analyses of catalytic C431. Solvent-accessible surface area (SASA) for C431 of the RING2 domain.

We observe that the Y143E variant’s C431 has the least exposure to the solvent. In contrast, the highest variation in the SASA is with the WT (Figure 7). This alludes to different mechanisms of activation by each of the mutations and potentially describes multiple routes of activation for small molecule activators in the future.

#### 3.4.2. Distance between the E2 Binding Site of RING1 and Parkin C431

In Parkin’s inactive state, the E2 binding site of RING1 and Parkin’s catalytic center are separated by a large distance, preventing Ub transfer to C431. For this purpose, we calculated the center-of-mass distance between the E2 binding site and C431. The distance in WT showed several trends that transitioned in a stepwise fashion; this indicates conformational variability and potentially several discrete states. The W403A mutation shows the overall greatest distance and variance in distance; perhaps it functions through the destabilization of the inactive state.

During our investigation of the conformational dynamics of Parkin, we found that the values for distance, RMSD, and SASA all underwent considerable shifts due to the different mutations, which likely play a substantial role in allowing relaxation of the auto-inhibited nature of the Parkin and, consequently, activation of its E3 Ub ligase capabilities.

### 3.5. Local Interaction Perturbations of the Parkin Variants

The individual variants discussed accomplish the abovementioned more global and regional changes by first altering local contacts, which then propagate the motions outward to other regions of the protein.

Y143 in WT Parkin fits nicely into a cavity making a cluster of pi-pi or hydrophobic-pi interactions with P132, A134, Y147, P159, P247, and I306. Many of these contacts are disrupted by the substitution for the non-aromatic/non-hydrophobic glutamate. Y143E repels away from many of these and makes intermittent hydrogen bonds with Y147 and/or R140.

V393 in WT Parkin is a hydrophobic residue on a helix that is partially exposed to solvent. It does not appear to make any native contacts. However, substituted for an aspartate, the helix twists in a different direction to expose the charged residue to solvent instead of pointing away; this twist disrupts the REP. The disrupted REP conformation appears to be partially stabilized by the increased propensity for the sidechain of R271 to form a hydrogen bond with R392.

A401 is also a hydrophobic residue on the same partially solvent-exposed helix and, therefore, tries to point toward the core of the protein. The small alanine is able to tuck nicely between RING0 and RING1 and makes hydrophobic interactions with I239 and V258. Substitution with the aspartate causes the charged residue to twist the helix outward for solvent exposure, again disrupting the REP. This disrupted conformation is partially stabilized by a hydrogen bond that occurs intermittently between D401 and the backbone of T237 or the backbone of I239.

W403 in WT Parkin fits well in a cavity at the junction of RING0, RING1, and RING2. It forms a cation-pi interaction with R234, as well as hydrophobic-pi interactions with several residues: A230, I236, V248, V258, and V465. Substitution with the much smaller and non-aromatic alanine disrupts most of these interactions and causes RING0 and RING1 to pull in more closely, pushing away the REP from RING1. The collapse of the cavity formerly occupied by the larger W403 is stabilized by altering the probability of a variety of interactions, such as hydrogen bonds between N232 and I236, V238 and A230 (backbones), R256 and V465, and R234 and A403. Additionally, the percentage of time that other nearby hydrophobic interactions form has been altered: V248 to V258, V250 to V465, and A230 to V250.

Overall, local changes to interactions due to the point substitutions have been observed. More importantly, these local changes propagate to larger-scale motions that deviate from the variants from WT. Since they are each unique substitutions, their local impact is also unique; however, they all disrupt the dynamical behavior of Parkin, and we believe these observed alterations are consistent with the disturbance of the auto-inhibited conformation and, ergo, uncover the initial progression to conformational transition.

## 4. Discussion and Conclusions

Capturing the full Parkin activation would require inordinately long unbiased simulations or, more likely, as in our previous study, using enhanced sampling techniques. From this prior study, we have determined that disruptions of the inactive conformation of Parkin are an apposite early indicator that activation is initiating. Given that our goal was to uncover the effects of these variants, known via experiments to result in hyperactive Parkin, on the initial stages of activation, the timeframe of our simulation observation is relevant. We chose to examine the initial differences that might lead to facilitated activation in unbiased simulations against variants that have previously been experimentally validated, as this is a computationally understudied mechanism with respect to Parkin.

Due to the popularity of AlphaFold, older modeling methods are consistently called into question. We compared our homology model to the one predicted by AlphaFold and chose to utilize our model. Notably, AlphaFold does not include structural zincs and would require post hoc addition of them; this is not difficult, but our model was complete with these constructed with proper coordination sites. The AlphaFold model also has an entirely unrealistic linker region. While the electron density for the linker region is absent in all X-ray crystal structures, this is indicative of mobility of the domain, rather than a complete lack of any secondary structural features; indeed, the sequence-based annotation of this region predicts that only residues 77–99 are disordered [57]. Perhaps under physiological conditions, the linker develops structural elements while retaining a level of mobility; however, we cannot rule out that the structuring of this region is an artifact of the modeling algorithm to generate a maximally stable model under the given conditions. We believe, in any case, that our observations of differences that might proceed to the initial activation of Parkin via a large-scale global conformation change are still appropriate, given our window of observation.

The most prevalent genetic cause of early-onset PD is represented by loss-of-function mutations in *PRKN*. The analysis of the Parkin E3 Ub ligase activity and structure thus far has been exemplary in comprehending the different mechanisms by which PD mutations interfere with Parkin activation or function. Our findings indicate that the various activating mutations (Y143E, A401D, V393D, and W403A) present in Parkin have a discernible impact on Parkin structure. This is achieved by inducing a conformational alteration in the inactive closed Parkin, as we observed through the utilization of a molecular dynamics simulation. These results also allude to different mechanisms of activation for each of the hyperactive variants and may prove valuable for subsequent experimental inquiries aiming to find the appropriate level of activation, with the goal of providing therapeutic benefits while still maintaining steady-state Parkin protein levels.

## Data Availability

Data and models will be made available upon request to the corresponding author.

## Supplementary Materials

The following supporting information can be downloaded at: https://www.mdpi.com/article/10.3390/biom14030365/s1, Figure S1: Global dynamical comparisons of Parkin variants; Figure S2: Structural network comparisons of Parkin variants; Figure S3: Probability of finding REP residue at the E2-ubl binding site of Ring1 domain for different mutation as well as for wild-type parkin; Figure S4: RING0-RING2 regional dynamical comparisons of Parkin variants; Figure S5: RING0-RING1 regional dynamical comparisons of Parkin variants.

## References

[1] Poewe W., Seppi K., Tanner C.M., Halliday G.M., Brundin P., Volkmann J., Schrag A.E., Lang A.E. (2017). Parkinson disease. Nat. Rev. Dis. Primers.

[2] Kitada T., Asakawa S., Hattori N., Matsumine H., Yamamura Y., Minoshima S., Yokochi M., Mizuno Y., Shimizu N. (1998). Mutations in the parkin gene cause autosomal recessive juvenile parkinsonism. Nature.

[3] Valente E.M., Abou-Sleiman P.M., Caputo V., Muqit M.M., Harvey K., Gispert S., Ali Z., Del Turco D., Bentivoglio A.R., Healy D.G. (2004). Hereditary early-onset Parkinson’s disease caused by mutations in PINK1. Science.

[4] Geisler S., Holmstrom K.M., Skujat D., Fiesel F.C., Rothfuss O.C., Kahle P.J., Springer W. (2010). PINK1/Parkin-mediated mitophagy is dependent on VDAC1 and p62/SQSTM1. Nat. Cell Biol..

[5] Matsuda N., Sato S., Shiba K., Okatsu K., Saisho K., Gautier C.A., Sou Y.S., Saiki S., Kawajiri S., Sato F. (2010). PINK1 stabilized by mitochondrial depolarization recruits Parkin to damaged mitochondria and activates latent Parkin for mitophagy. J. Cell Biol..

[6] Narendra D.P., Jin S.M., Tanaka A., Suen D.F., Gautier C.A., Shen J., Cookson M.R., Youle R.J. (2010). PINK1 is selectively stabilized on impaired mitochondria to activate Parkin. PLoS Biol..

[7] Vives-Bauza C., Zhou C., Huang Y., Cui M., de Vries R.L., Kim J., May J., Tocilescu M.A., Liu W., Ko H.S. (2010). PINK1-dependent recruitment of Parkin to mitochondria in mitophagy. Proc. Natl. Acad. Sci. USA.

[8] Kane L.A., Lazarou M., Fogel A.I., Li Y., Yamano K., Sarraf S.A., Banerjee S., Youle R.J. (2014). PINK1 phosphorylates ubiquitin to activate Parkin E3 ubiquitin ligase activity. J. Cell Biol..

[9] Kazlauskaite A., Kondapalli C., Gourlay R., Campbell D.G., Ritorto M.S., Hofmann K., Alessi D.R., Knebel A., Trost M., Muqit M.M. (2014). Parkin is activated by PINK1-dependent phosphorylation of ubiquitin at Ser65. Biochem. J..

[10] Koyano F., Okatsu K., Kosako H., Tamura Y., Go E., Kimura M., Kimura Y., Tsuchiya H., Yoshihara H., Hirokawa T. (2014). Ubiquitin is phosphorylated by PINK1 to activate parkin. Nature.

[11] Iguchi M., Kujuro Y., Okatsu K., Koyano F., Kosako H., Kimura M., Suzuki N., Uchiyama S., Tanaka K., Matsuda N. (2013). Parkin-catalyzed ubiquitin-ester transfer is triggered by PINK1-dependent phosphorylation. J. Biol. Chem..

[12] Kondapalli C., Kazlauskaite A., Zhang N., Woodroof H.I., Campbell D.G., Gourlay R., Burchell L., Walden H., Macartney T.J., Deak M. (2012). PINK1 is activated by mitochondrial membrane potential depolarization and stimulates Parkin E3 ligase activity by phosphorylating Serine 65. Open Biol..

[13] Shiba-Fukushima K., Imai Y., Yoshida S., Ishihama Y., Kanao T., Sato S., Hattori N. (2012). PINK1-mediated phosphorylation of the Parkin ubiquitin-like domain primes mitochondrial translocation of Parkin and regulates mitophagy. Sci. Rep..

[14] Truban D., Hou X., Caulfield T.R., Fiesel F.C., Springer W. (2017). PINK1, Parkin, and Mitochondrial Quality Control: What can we Learn about Parkinson’s Disease Pathobiology?. J. Parkinsons Dis..

[15] Wenzel D.M., Lissounov A., Brzovic P.S., Klevit R.E. (2011). UBCH7 reactivity profile reveals parkin and HHARI to be RING/HECT hybrids. Nature.

[16] Chaugule V.K., Burchell L., Barber K.R., Sidhu A., Leslie S.J., Shaw G.S., Walden H. (2011). Autoregulation of Parkin activity through its ubiquitin-like domain. EMBO J..

[17] Riley B.E., Lougheed J.C., Callaway K., Velasquez M., Brecht E., Nguyen L., Shaler T., Walker D., Yang Y., Regnstrom K. (2013). Structure and function of Parkin E3 ubiquitin ligase reveals aspects of RING and HECT ligases. Nat. Commun..

[18] Spratt D.E., Martinez-Torres R.J., Noh Y.J., Mercier P., Manczyk N., Barber K.R., Aguirre J.D., Burchell L., Purkiss A., Walden H. (2013). A molecular explanation for the recessive nature of parkin-linked Parkinson’s disease. Nat. Commun..

[19] Trempe J.F., Fon E.A. (2013). Structure and Function of Parkin, PINK1, and DJ-1, the Three Musketeers of Neuroprotection. Front. Neurol..

[20] Wauer T., Komander D. (2013). Structure of the human Parkin ligase domain in an autoinhibited state. EMBO J..

[21] Caulfield T.R., Fiesel F.C., Moussaud-Lamodière E.L., Dourado D.F.A.R., Flores S.C., Springer W. (2014). Phosphorylation by PINK1 Releases the UBL Domain and Initializes the Conformational Opening of the E3 Ubiquitin Ligase Parkin. PLoS Comput. Biol..

[22] Caulfield T.R., Fiesel F.C., Springer W. (2015). Activation of the E3 ubiquitin ligase Parkin. Biochem. Soc. Trans..

[23] Zhou C., Huang Y., Shao Y., May J., Prou D., Perier C., Dauer W., Schon E.A., Przedborski S. (2008). The kinase domain of mitochondrial PINK1 faces the cytoplasm. Proc. Natl. Acad. Sci. USA.

[24] Kazlauskaite A., Martinez-Torres R.J., Wilkie S., Kumar A., Peltier J., Gonzalez A., Johnson C., Zhang J., Hope A.G., Peggie M. (2015). Binding to serine 65-phosphorylated ubiquitin primes Parkin for optimal PINK1-dependent phosphorylation and activation. EMBO Rep..

[25] Sauve V., Lilov A., Seirafi M., Vranas M., Rasool S., Kozlov G., Sprules T., Wang J., Trempe J.F., Gehring K. (2015). A Ubl/ubiquitin switch in the activation of Parkin. EMBO J..

[26] Wauer T., Simicek M., Schubert A., Komander D. (2015). Mechanism of phospho-ubiquitin-induced PARKIN activation. Nature.

[27] Wauer T., Swatek K.N., Wagstaff J.L., Gladkova C., Pruneda J.N., Michel M.A., Gersch M., Johnson C.M., Freund S.M., Komander D. (2015). Ubiquitin Ser65 phosphorylation affects ubiquitin structure, chain assembly and hydrolysis. EMBO J..

[28] Condos T.E., Dunkerley K.M., Freeman E.A., Barber K.R., Aguirre J.D., Chaugule V.K., Xiao Y., Konermann L., Walden H., Shaw G.S. (2018). Synergistic recruitment of UbcH7~Ub and phosphorylated Ubl domain triggers parkin activation. EMBO J..

[29] Gladkova C., Maslen S.L., Skehel J.M., Komander D. (2018). Mechanism of parkin activation by PINK1. Nature.

[30] Ordureau A., Paulo J.A., Zhang W., Ahfeldt T., Zhang J., Cohn E.F., Hou Z., Heo J.M., Rubin L.L., Sidhu S.S. (2018). Dynamics of PARKIN-Dependent Mitochondrial Ubiquitylation in Induced Neurons and Model Systems Revealed by Digital Snapshot Proteomics. Mol. Cell.

[31] Sauve V., Sung G., Soya N., Kozlov G., Blaimschein N., Miotto L.S., Trempe J.F., Lukacs G.L., Gehring K. (2018). Mechanism of parkin activation by phosphorylation. Nat. Struct. Mol. Biol..

[32] Trempe J.F., Sauve V., Grenier K., Seirafi M., Tang M.Y., Menade M., Al-Abdul-Wahid S., Krett J., Wong K., Kozlov G. (2013). Structure of parkin reveals mechanisms for ubiquitin ligase activation. Science.

[33] Ge P., Dawson V.L., Dawson T.M. (2020). PINK1 and Parkin mitochondrial quality control: A source of regional vulnerability in Parkinson’s disease. Mol. Neurodegener..

[34] Martinez A., Lectez B., Ramirez J., Popp O., Sutherland J.D., Urbe S., Dittmar G., Clague M.J., Mayor U. (2017). Quantitative proteomic analysis of Parkin substrates in Drosophila neurons. Mol. Neurodegener..

[35] Tang M.Y., Vranas M., Krahn A.I., Pundlik S., Trempe J.F., Fon E.A. (2017). Structure-guided mutagenesis reveals a hierarchical mechanism of Parkin activation. Nat. Commun..

[36] Yi W., MacDougall E.J., Tang M.Y., Krahn A.I., Gan-Or Z., Trempe J.F., Fon E.A. (2019). The landscape of Parkin variants reveals pathogenic mechanisms and therapeutic targets in Parkinson’s disease. Hum. Mol. Genet..

[37] de Bie P., Ciechanover A. (2011). Ubiquitination of E3 ligases: Self-regulation of the ubiquitin system via proteolytic and non-proteolytic mechanisms. Cell Death Differ..

[38] Watzlawik J.O., Fiesel F.C., Fiorino G., Bustillos B.A., Baninameh Z., Markham B.N., Hou X., Hayes C.S., Bredenberg J.M., Kurchaba N.W. (2023). Basal activity of PINK1 and PRKN in cell models and rodent brain. Autophagy.

[39] Stevens M.U., Croteau N., Eldeeb M.A., Antico O., Zeng Z.W., Toth R., Durcan T.M., Springer W., Fon E.A., Muqit M.M. (2023). Structure-based design and characterization of Parkin-activating mutations. Life Sci. Alliance.

[40] Land H., Humble M.S. (2018). YASARA: A Tool to Obtain Structural Guidance in Biocatalytic Investigations. Methods Mol. Biol..

[41] Fiesel F.C., Fricova D., Hayes C.S., Coban M.A., Hudec R., Bredenberg J.M., Broadway B.J., Markham B.N., Yan T., Boneski P.K. (2022). Substitution of PINK1 Gly411 modulates substrate receptivity and turnover. Autophagy.

[42] Puschmann A., Fiesel F.C., Caulfield T.R., Hudec R., Ando M., Truban D., Hou X., Ogaki K., Heckman M.G., James E.D. (2017). Heterozygous PINK1 p.G411S increases risk of Parkinson’s disease via a dominant-negative mechanism. Brain.

[43] Polak B., Ribière G. (1969). Note sur la convergence des méthodes de directions conjuguées. Rev. Française D’informatique Et De Rech. Opérationnelle Série Rouge.

[44] Berendsen H.J.C., Postma J.P.M., van Gunsteren W.F., DiNola A., Haak J.R. (1984). Molecular dynamics with coupling to an external bath. J. Chem. Phys..

[45] Parrinello M., Rahman A. (1981). Polymorphic transitions in single crystals: A new molecular dynamics method. J. Appl. Phys..

[46] McGibbon R.T., Beauchamp K.A., Harrigan M.P., Klein C., Swails J.M., Hernandez C.X., Schwantes C.R., Wang L.P., Lane T.J., Pande V.S. (2015). MDTraj: A Modern Open Library for the Analysis of Molecular Dynamics Trajectories. Biophys. J..

[47] Humphrey W., Dalke A., Schulten K. (1996). VMD: Visual molecular dynamics. J. Mol. Graph..

[48] Janson G., Zhang C., Prado M.G., Paiardini A. (2017). PyMod 2.0: Improvements in protein sequence-structure analysis and homology modeling within PyMOL. Bioinformatics.

[49] Schrödinger (2022). Biologics Suite.

[50] Caulfield T., Devkota B. (2012). Motion of transfer RNA from the A/T state into the A-site using docking and simulations. Proteins.

[51] Caulfield T., Medina-Franco J.L. (2011). Molecular dynamics simulations of human DNA methyltransferase 3B with selective inhibitor nanaomycin A. J. Struct. Biol..

[52] Caulfield T.R. (2011). Inter-ring rotation of apolipoprotein A-I protein monomers for the double-belt model using biased molecular dynamics. J. Mol. Graph. Model..

[53] Caulfield T.R., Devkota B., Rollins G.C. (2011). Examinations of tRNA Range of Motion Using Simulations of Cryo-EM Microscopy and X-Ray Data. J. Biophys..

[54] Kayode O., Wang R., Pendlebury D.F., Cohen I., Henin R.D., Hockla A., Soares A.S., Papo N., Caulfield T.R., Radisky E.S. (2016). An Acrobatic Substrate Metamorphosis Reveals a Requirement for Substrate Conformational Dynamics in Trypsin Proteolysis. J. Biol. Chem..

[55] Girvan M., Newman M.E. (2002). Community structure in social and biological networks. Proc. Natl. Acad. Sci. USA.

[56] Eargle J., Luthey-Schulten Z. (2012). NetworkView: 3D display and analysis of protein.RNA interaction networks. Bioinformatics.

[57] Piovesan D., Necci M., Escobedo N., Monzon A.M., Hatos A., Micetic I., Quaglia F., Paladin L., Ramasamy P., Dosztanyi Z. (2021). MobiDB: Intrinsically disordered proteins in 2021. Nucleic Acids Res..

