# Supplementary material for: In Silico Investigation of Parkin-Activating Mutations Using Simulations and Network Modeling
Source: Biomolecules. 2024 Mar 19;14(3):365. doi: 10.3390/biom14030365 (PMC10968616; doi:10.3390/biom14030365)
Supplement: Supplementary file 1 [file biomolecules-14-00365-s001.zip › supplemental figures and data ParkinMutants-Penultimate-MAC-17Jan24_FF_v2.pdf]

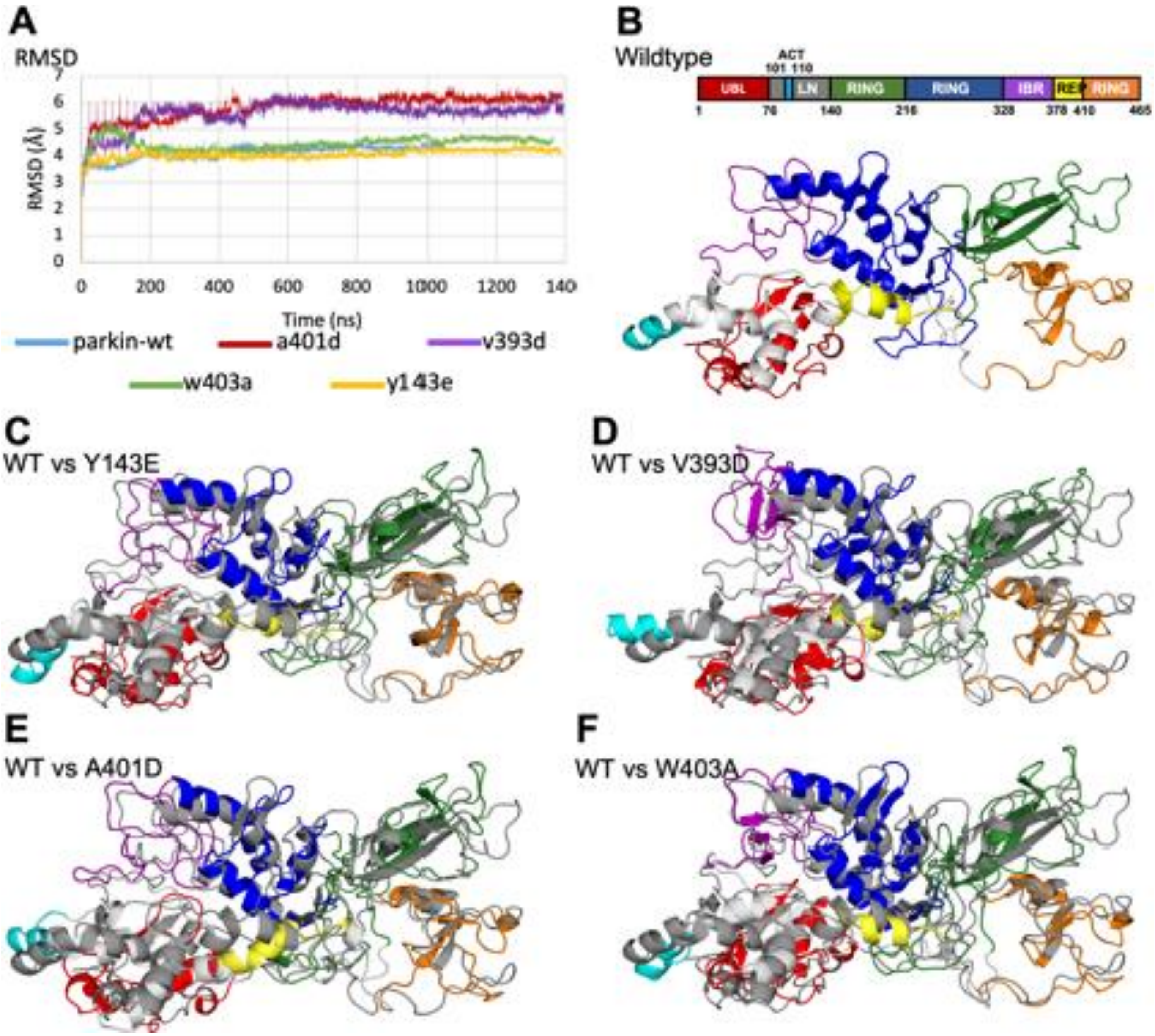

Figure S1. Global dynamical comparisons of Parkin variants. (A) RMSD of the entire Parkin protein over time throughout the course of the simulation. **(B)** Domain map of the Parkin Wildtype (WT). **(C)** Overlap of the WT and Y143E. **(D)** Overlap of the WT and V393D. **(E)** Overlap of the WT and A401D. **(F)** Overlap of the WT and W403A.

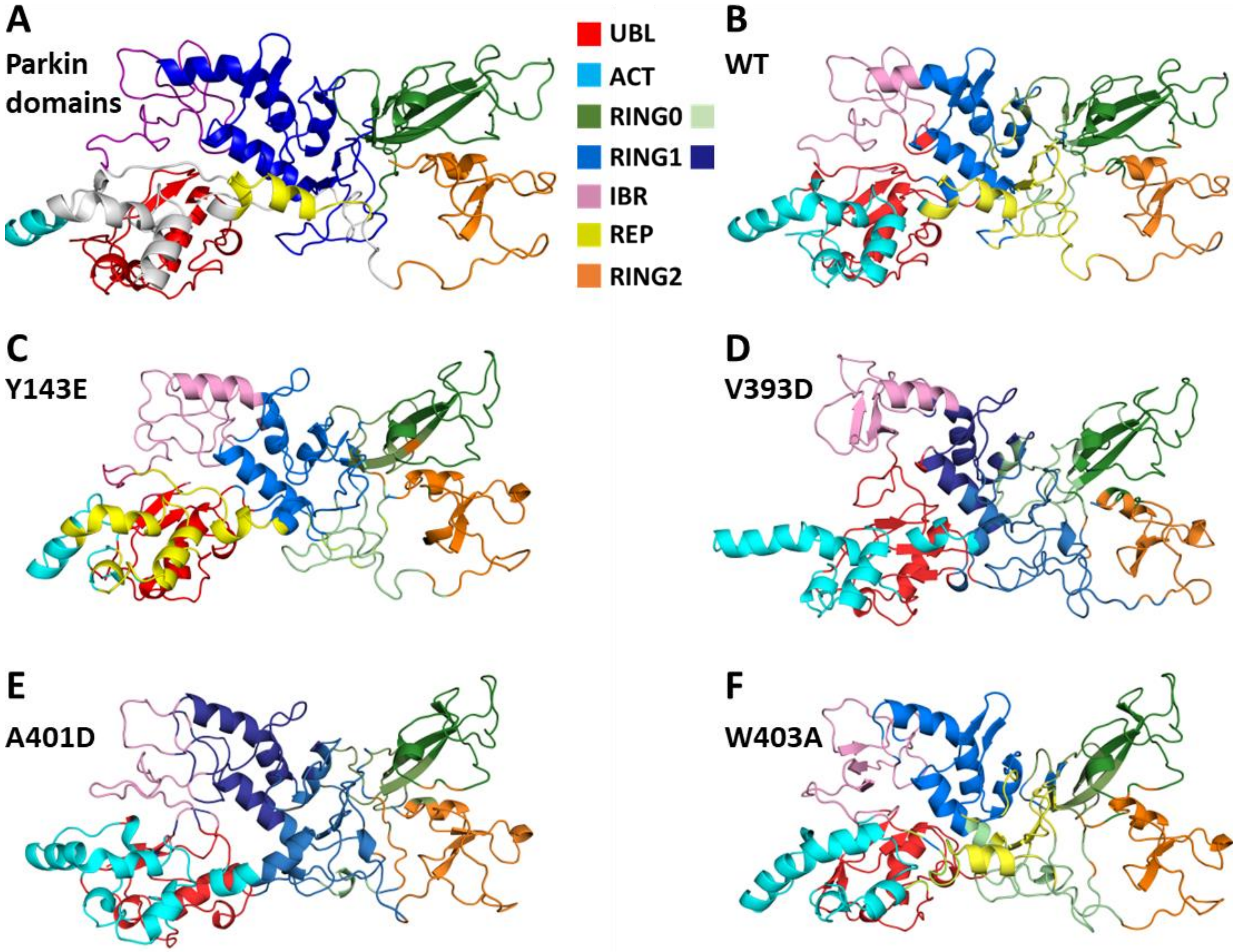

Figure S2. Structural network comparisons of Parkin variants. (A) 3D domain map of Parkin. **(B)** Dynamical network of WT Parkin, colored to correspond to the domain map. **(C)** Dynamical network of Y143E. **(D)** Dynamical network of V393D. **(E)** Dynamical network of A401D. **(F)** Dynamical network W403A.

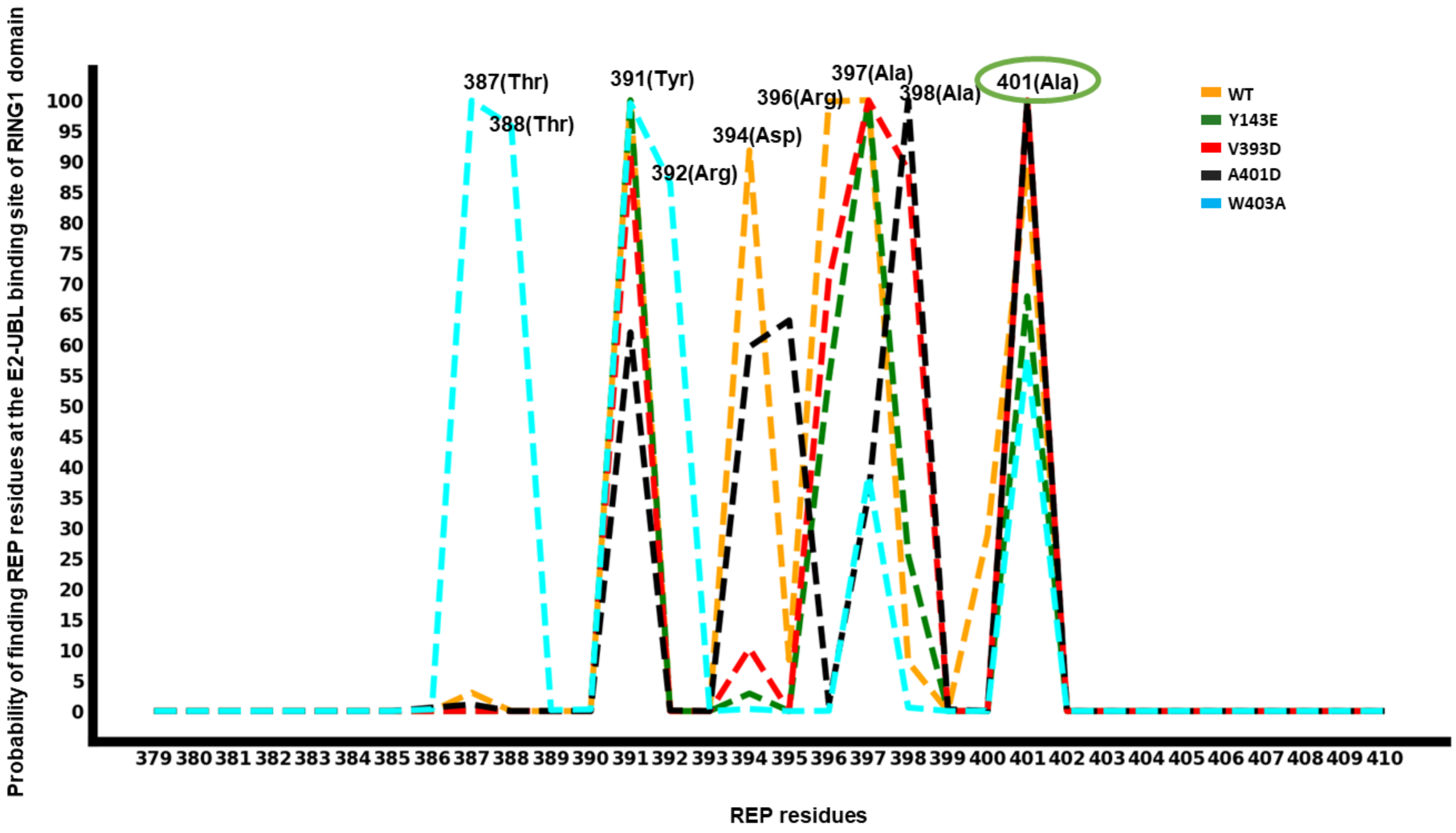

Figure S3. Global dynamical comparisons of Parkin variants. Probability of finding REP residue at the E2-UBL binding site of RING1 domain for different mutations, as well as for WT Parkin.

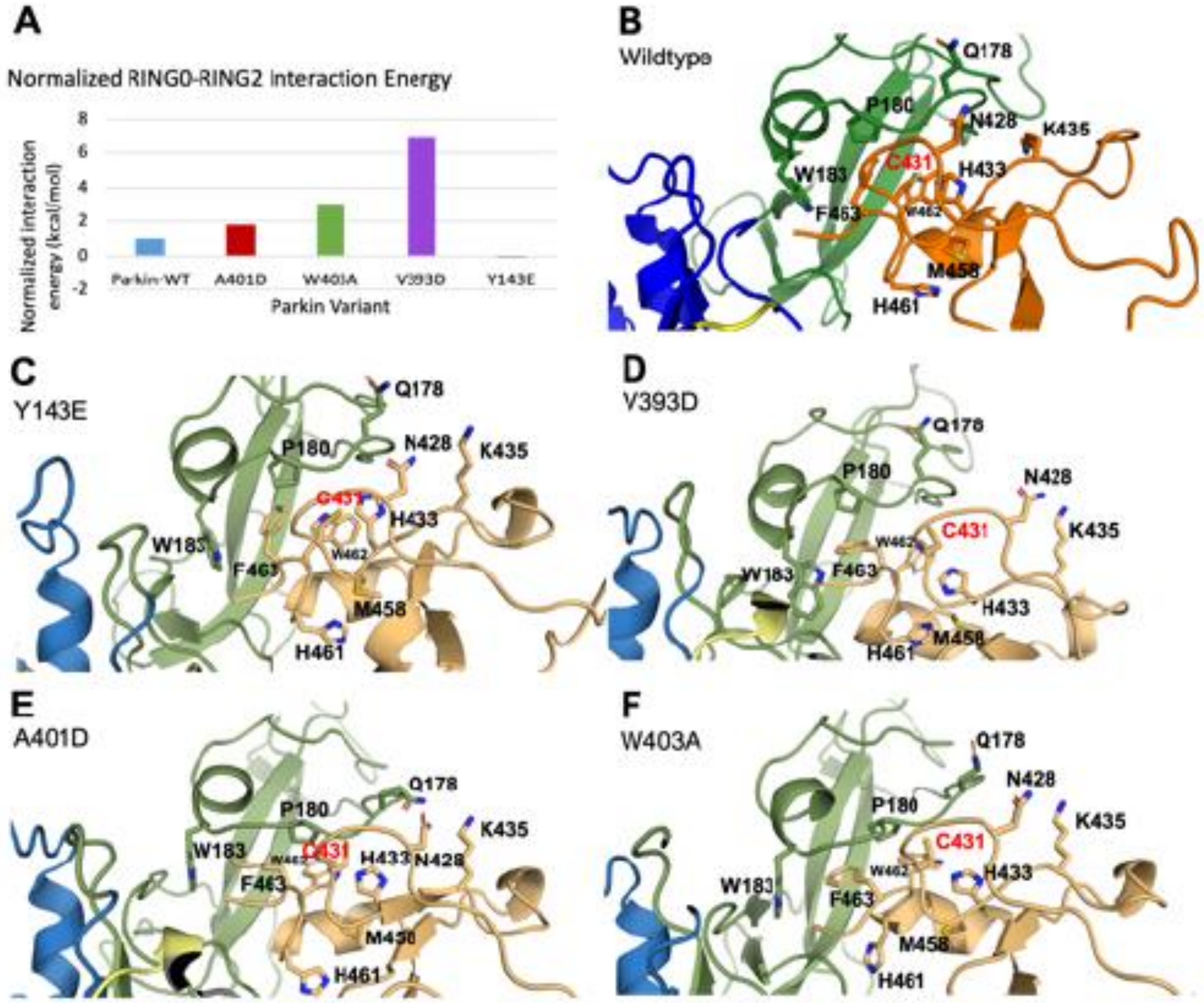

Figure S4. RING0-RING2 regional dynamical comparisons of Parkin variants. Domain-Domain interactions between the **(A)** RING0 and RING2 domains over time throughout the course of the simulation. Close up of the RING0-RING2 interactions for **(B)** WT, **(C)** Y143E, **(D)** V393D, **(E)** A401D, and **(F)** W403A.

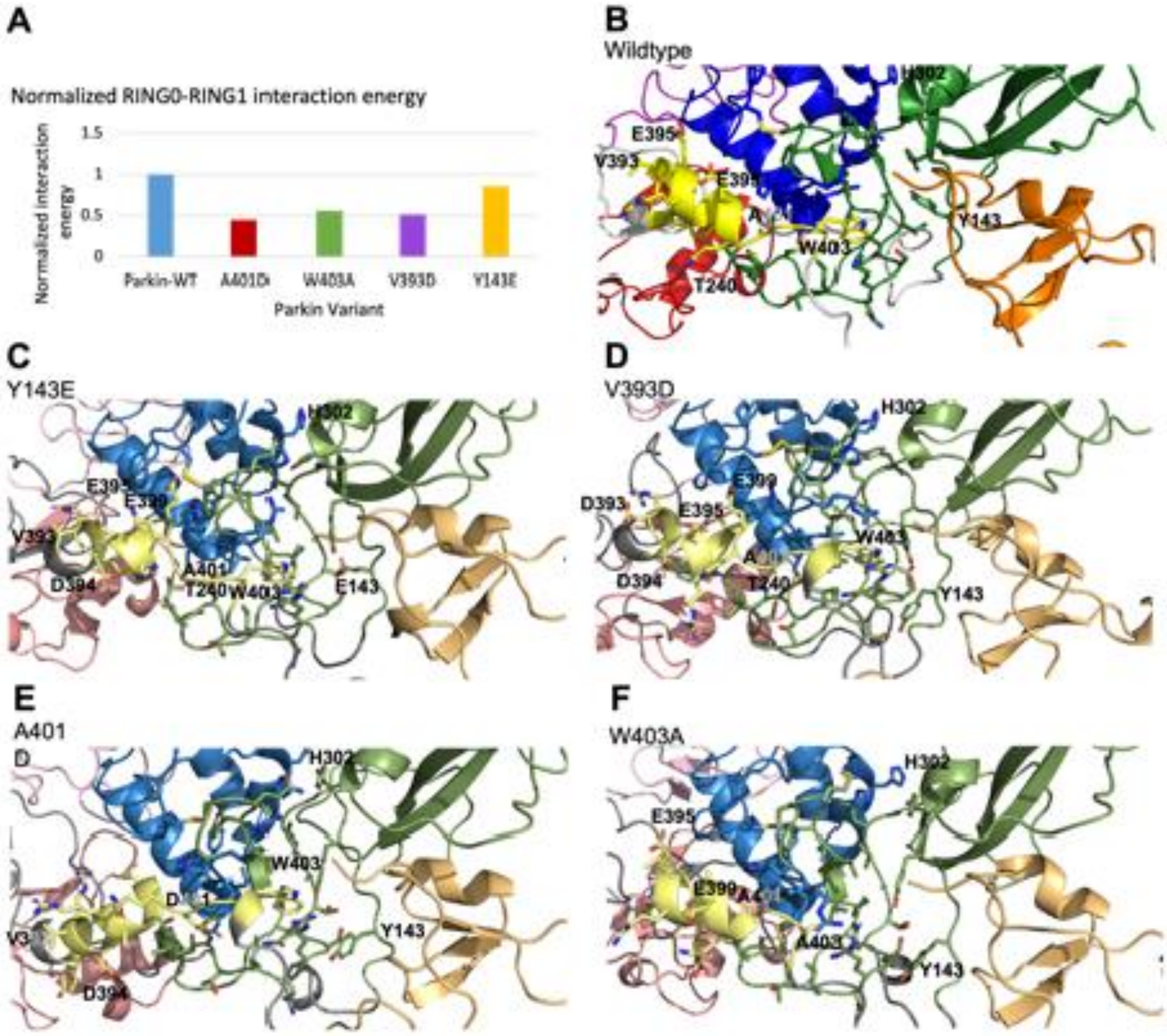

Figure S5. RING0-RING1 regional dynamical comparisons of Parkin variants. Domain-Domain interactions between the **(A)** RING0 and RING1 domains over time throughout the course of the simulation. Close up of the RING0-RING1 interactions for **(B)** WT, **(C)** Y143E, **(D)** V393D, **(E)** A401D, and **(F)** W403A.
